# From Children to Adults: Motor Performance across the Life-Span

**DOI:** 10.1371/journal.pone.0038830

**Published:** 2012-06-18

**Authors:** Jonas S. R. Leversen, Monika Haga, Hermundur Sigmundsson

**Affiliations:** 1 Department of Psychology, Norwegian University of Science and Technology, Trondheim, Norway; 2 Department of Physiotherapy, Faculty of Health and Social Work, Sør-Trøndelag University College, Trondheim, Norway; University Medical Center Groningen UMCG, Netherlands

## Abstract

The life-span approach to development provides a theoretical framework to examine the general principles of life-long development. This study aims to investigate motor performance across the life span. It also aims to investigate if the correlations between motor tasks increase with aging. A cross-sectional design was used to describe the effects of aging on motor performance across age groups representing individuals from childhood to young adult to old age. Five different motor tasks were used to study changes in motor performance within 338 participants (7–79 yrs). Results showed that motor performance increases from childhood (7–9) to young adulthood (19–25) and decreases from young adulthood (19–25) to old age (66–80). These results are mirroring results from cognitive research. Correlation increased with increasing age between two fine motor tasks and two gross motor tasks. We suggest that the findings might be explained, in part, by the structural changes that have been reported to occur in the developing and aging brain and that the theory of Neural Darwinism can be used as a framework to explain why these changes occur.

## Introduction

The perspective of life span development provides a framework to study how and why individual changes occur across the whole life-course. Taking an ecological approach, individuals are looked upon as constantly adapting to the environment and thus ontogenetic change occurs as a consequence of interplay between the environment and the individual. Baltes and Lindenberger [1] describes that the perspective of life span development aims to *“obtain knowledge about general principles of life-long development, about inter-individual differences and similarities in development, as well as about the degree and conditions of individual plasticity or modifiability of development” (p.611).*

Most developmental research has either focused on changes in early development or on aging, and knowledge about the general principles of life long development is still insufficient. To delineate general principles of life-span development, longitudinal studies that measure the same individuals from childhood to old age is preferable. However, such longitudinal studies are time consuming and the few that exist often follow individuals from young adulthood to old age [2]. Use of cross-sectional samples with different age groups has been the most common study design in this kind of research. In the following; the term ‘early development’ is used to describe changes from birth to young adulthood, while ‘late development’ is used to describe the changes that occur from young adulthood to old age. The following section will give a presentation of theories concerning general patterns of life-span development and *Neural Darwinism*.

### General patterns of early development

Early development is characterized by an increase in performance, such as decrease in reaction time, and an increase in processing speed and intelligence [3]. Structurally, grey matter volume decreases and white matter volume increases [4], [5].

### General patterns of late development

Late development is characterized by differential patterns of change and stability. There is a linear reduction of performance in tasks that are dependent on speed, such as processing speed or finger tapping [6], [7], [8]. Semantic memory is relatively stable up to the age of 65 [2], [9]. Structurally, there is a reduction of grey matter [10] and a reduction of white matter volume [11]. Functional imaging has shown that task specific activations in cognitive tasks become more global in ‘high performing old adults’ suggesting that this group counteracted age-related neural decline through a plastic reorganization of neurocognitive networks (compensation hypothesis) [12], [13]. The results also show that ‘low-performing older adults’ recruited a similar network as young adults but used it inefficiently compared to the younger subjects. With a cross-sectional design, Sowell et al. [14] studied structural changes across the life-span. Findings indicated a reduction of grey matter volume following a nonlinear reduction from childhood to old adulthood, while white matter volume follows an inverted U shape, with low white matter volume in both children and old adults. Studies indicate a positive relationship between large white matter volume and processing speed [15], additionally decrease in white matter volume is associated with poor motor function [16].

### Neural Darwinism and development

One biological theory that maintains the ecological approach is neural Darwinism (ND), or the theory of neuronal group selection [17], [18]. This concept has been translated to the domain of human development to understand the probabilistic epigenetic nature of the entire developing process [19]–[23]. Building on Darwin's principles, Edelman [17], [18] argues that the process of development can be explained as a process of selection that takes place inside the nervous system. One of the principle properties of the developing brain is its repertoires of neuronal groups. These units are collections of hundreds and thousands of strongly interconnected neurones and are considered to be the basic functional units or units of selection in the brain. The structural variability or ‘neural diversity’ that these units represent can give rise to many different outputs. Given the enormous possibility for variation, it is impossible to characterize the neurological connections within our brain as predetermined. On the contrary, the possibilities of variation suggest that selection based on experience occurs, i.e., that certain links – neuronal groups – can be strengthened if they are actively used. According to ND, development is the result of a complex interaction between genetic information and environmental factors. Experience increases connections within specific areas of the brain and strengthens the neural group which is used.

There is empirical support for Edelman's claims. That activated neurons in close proximity wire together has been known since the 70's when long term potentiation (LTP) was first demonstrated in rabbits [24]. The existence of neural groups has recently been discovered, and research indicates that groups of neurons called central pattern generators (CPG) are capable of generating locomotion activity [25], [26], and that distinct neural groups in the visual system handle different aspects of visual stimuli [27]. The formation of some neural groups is probably genetically determined but research indicates that much of synapto-genesis and structural change are dependent on post- natal experience [28], [29]. For example, neural groups in rats, called grid cells, can adequately represent a map of the environment, and these maps are malleable to changes in the environment [30]. From this line of thinking it follows that different tasks could be sub served by distinct neural groups.

### From general patterns to general principles

If the changes described earlier are to be considered general patterns of life-span development then they should be apparent in other modalities as well. Within the motor domain longitudinal and cross-sectional research are sparse [31]. However, existing research indicates that motor performance becomes better from childhood to young adulthood [32] and decreases in old age [33], [34]. This study aims to investigate motor performance across the life span, exploring if the patterns of motor performance over the life-span was similar to those established by research in cognitive domain.

In search of general principles we wanted to explore if the principles suggested by Neural Darwinism could explain the changes in late development. Neural groups should always adhere to Darwinian principles. As already described, grey matter volume decreases throughout the life span while white matter volume follows an inverted U shape. Therefore, in old age it is plausible to assume that fewer neural groups would be available for task performance.

If different neural groups are responsible for distinct types of processing, low correlations between similar tasks for young individuals could be expected due to an abundance of neural groups. Contrary, with increased age, correlations between similar tasks should increase. High correlations between cognitive tasks are associated with aging [35], even when reductions in sensory acuity are accounted for [36]. To investigate if increasing correlations between tasks were evident in the motor domain; two similar fine motor tasks and two gross motor tasks were selected.

In summary, we addressed the following questions:

1. Are the patterns of lifespan development established by cognitive research evident in the motor domain?
  We predicted that we would find an increase in motor- performance from childhood to young adulthood and a decrease in motor- performance from young adulthood to old age.
2. Are the correlations between motor tasks increasing with age?
  We predicted that correlations should be low in the youngest group and increase with increasing age.

## Methods

A cross-sectional design was used to describe the effects of aging on motor performance across age groups representing individuals from childhood to young adult to old age.

### Participants

338 participants between 7 and 79 years of age completed assessment of five different motor tasks. Children from 7–9 years (N = 173) were randomly selected from two mainstream primary schools. The entire sample reflected the population of children attending schools in these areas and included children in a wide range of socio-economic backgrounds. No child had any behavioural, neurological or orthopaedic problem or any reported history of learning difficulties that would qualify as exclusions criteria for this study.

The parents of the children were given written information of the purposes of the study and had given their written consent. The adults (N = 165) participants were randomly selected from a group of visitors to a Government building in Trondheim, Norway. The adult participants were given written information of the purpose of the study and had given their written consent. All the participants had no primary uncorrected visual deficit; no medical condition that might interfere with their ability to carry out the five motor tasks. They were instructed to use either glasses or contact lenses if they usually wore them. The participants were divided into age-groups based on chronological age. The children were all put in the youngest group. The adults were divided so that mean age of the groups were a decade apart. There were seven age-groups; 7–9, 19–25, 26–35, 36–45, 46–55, 56–65, and 66–80. The number of participants and mean age for groups are presented in Table 1.

**Table 1:** Mean age for the age groups and raw scores for the motor tasks.

Age groups	N	Age		PB		BB		TBT		HTW		W/R	
		Mean (s)	(SD)	Mean (s)	SD	Mean (s)	SD	Mean (s)	SD	Mean (s)	SD	Mean (s)	SD
7–9	173	7,36	(0,56)	34,57	6,62	20,02	4,50	63,74	36,01	21,88	7,13	6,12	1,06
19–25	87	21,87	(1,61)	18,40	2,00	10,50	1,61	23,33	15,84	8,53	1,85	4,60	0,41
26–35	31	30,13	(3,04)	18,76	2,57	10,72	1,61	22,03	12,65	8,25	1,89	5,07	0,68
36–45	16	39,63	(2,92)	20,73	3,63	12,07	2,40	25,50	18,03	8,08	1,28	6,02	1,05
46–55	12	50,92	(3,42)	21,15	1,93	12,24	1,54	25,35	12,50	8,40	1,66	6,47	1,28
56–65	13	60,69	(3,59)	23,30	2,46	13,55	2,12	28,07	14,89	9,50	2,94	6,98	1,30
66–80	6	75,17	(2,79)	27,96	2,53	14,69	1,63	28,12	22,21	14,37	5,74	9,66	3,46

PB: Placing Bricks, BB: Building Bricks, TBT: Throwing a bean bag at a target, HTW: Heel to toe walking, W/R: Walking running in slopes.

### Measures of motor performance

#### Test of Motor Competence (TMC)

TMC was designed to test general motor competence [37]. It is standardizes test battery that provides a quantitative evaluation of motor competence for tasks of daily life across a wide range of motor skills. This makes it possible to investigate motor competence as a function of age. The TMC consist of five different tasks: two tasks based on manual dexterity, a hand-eye coordination task and two dynamic balance tasks. Except for “throwing bean bag at a target” that uses distance, the measure is time to completion. Correlation between TMC and MABC are 0.51 for 7–8 years old (mean age 7.89, SD 0.54) Norwegian children (N = 70).

The five motor tasks are described below:

#### Placing Bricks (PB)

18 square-shaped duplo™ bricks are to be placed on a duplo board (Which has room for 3×6 bricks) as fast as possible The participant is seated at a table and are given a practice run before the actual testing.

#### Building Bricks (BB)

12 square-shaped duplo™ bricks are used to build a tower as fast as possible. The participant holds one brick in one hand and one brick in the other. At a signal the participant assembles the bricks together one after one until all 12 have been put together. Neither of the arms are allowed to rest on the table. The bricks should be held in the air all the time.

#### Throwing Bean Bag at Target (TBT)

The target (diameter = 2 cm) is situated on the floor (marked by colored tape) 2 meters from the participant. The aim is to hit the target. The measure is the mean distance from the target in three consecutive throws (under or over arm throw).

#### Heel to toe walking (HTW)

This task is often called the ‘Tandem walking test’ and is consider to be a measure of dynamic balance capabilities. Participant are required to walk down a straight line (4, 5 m) as fast as they can placing their heel against the toes of the foot in each step.

#### Walking/Running in Slopes (W/R)

This task is also known as ‘The figure of eight test’. The participant starts at the starting point. When a signal is given the participant walks/runs as fast as possible in a figure of 8 around two marked lines (1 meter in width). Line 1 is 1 meter from the starting point and line 2 is 5.5 meter from the starting point. If the participant starts to go on the right side of line 1 – the subject will go to the left side of line 2, turn around, and go back on the right side of line 2 and left side line 1 – and over the starting point. The time is stopped when the participants arrives the starting point. The subject can choose which direction they go. The participants were wearing suitable shoes.

### Procedure

The study was performed in accordance with the Declaration of Helsinki. Ethical approval for this study was granted by the Norwegian Social Science Data Service.

Before data were gathered, participants and parents (children's group) were given written information about the study. Written permission was obtained from the participants and parents or guardian before involvement in the study. Identification numbers were used to maintain data confidentiality.

Children were assessed on motor performance in a quiet room during normal school hours, the adult participants were measured in a quiet room at the University Campus. All the participants were tested individually by the corresponding author. Each test item was explained and demonstrated before the participants started. Participants were given verbal encouragement and support throughout the testing procedure.

### Data reduction and analysis

The data were analyzed in SPSS (version 15), after first screening the data for entry errors. The occurrence of missing data was low (less than 5%) and was treated by listwise deletion. Raw scores for the age- groups are shown in table 1. As task three uses distance as measurement, task scores were transformed into standardized scores (z-scores) for the whole sample (N = 338). A total test score of motor performance (TS) was calculated for each individual by taking the sum of the z-scores for the five tasks. One-way ANOVA was used to analyze effect of age on motor performance (question 1). Post-hoc Bonferroni was used to analyze the difference between age-groups. Correlation between the two fine motor tasks and two gross motor tasks in question 2 were analyzed by Pearson correlation test. Statistical significance was set at P<.05.

## Results

**Figure 1:**
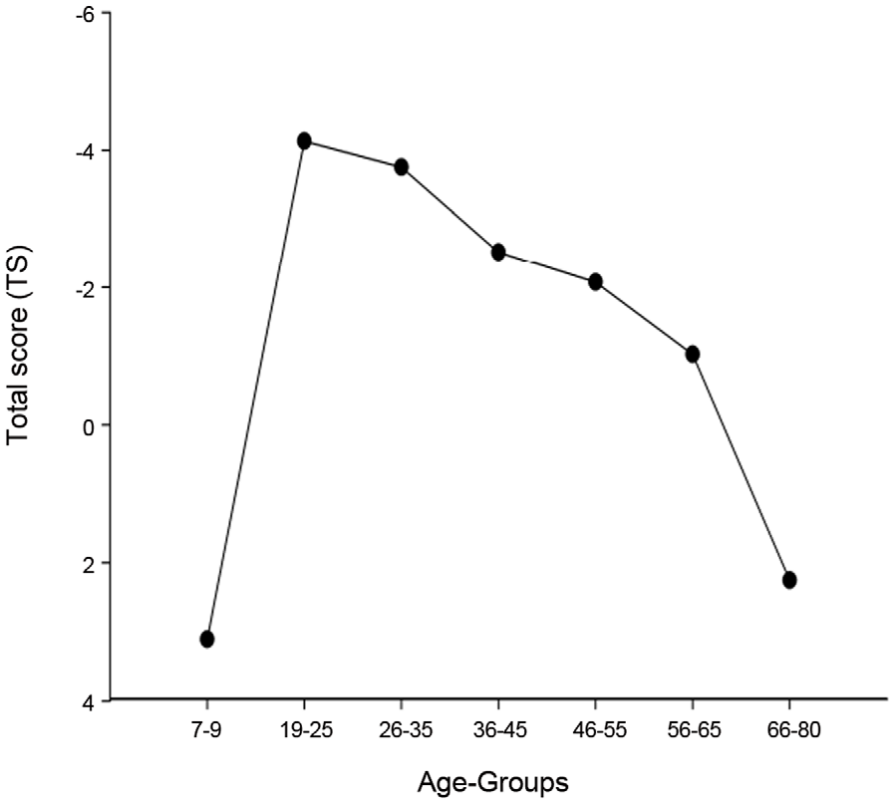
Total score for motor performances for all age-groups. Negative values indicate better performance.

The means and the standard deviations for age and the raw scores for the 5 different motor tasks for each age group are shown in table 1. The total score (TS) (the sum of the z-scores) for each age group is shown in fig. 1. A one way ANVOVA showed a significant main effect for age – group on motor competence F (6,316) = 172, 01, p<.001. A post- hoc Bonferroni analysis revealed that the youngest group (7–9) performed worse than the older age groups (p<.001) except for the oldest group (66–80). The oldest group performed worse than the younger age-groups except the 7–9 age group. No significant differences were detected between the middle aged groups, when divided in age spans of a decade. However, if the age groups were expanded to two or more decades significant differences were found.

**Figure 2:**
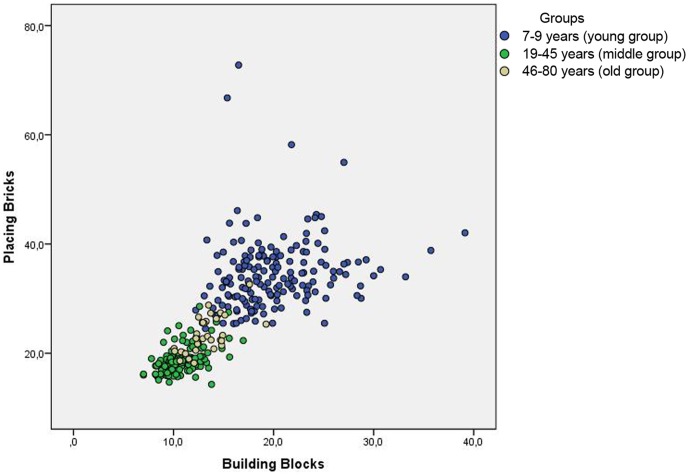
Correlation between Placing Bricks and Building Bricks for the three age groups (1) 7–9 years (young group, N = 173), (2) 19–45 (middle group, N = 134), (3) 46–80 years (old group, N = 31).

**Figure 3:**
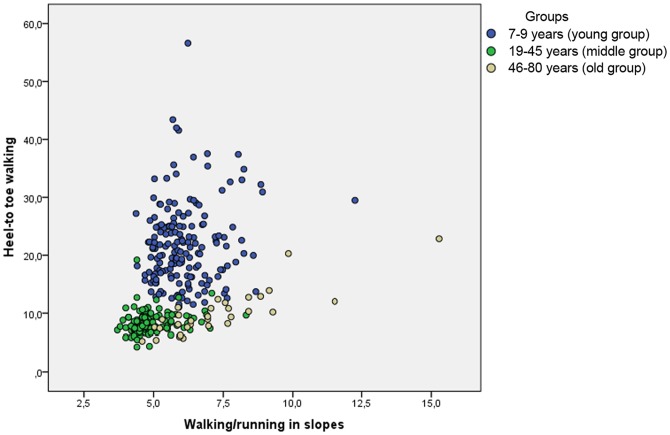
Correlation between Heel-to toe walking and Walking/running in slopes for the three age groups (1) 7–9 years (young group, N = 173), (2) 19–45 (middle group, N = 134), (3) 46–80 years (old group, N = 31).

Two fine motor tasks and two gross motor tasks with high task similarity were selected to investigate the relationship between tasks with similar motor requirements; placing bricks (PB), building bricks (BB), heel to toe walking (HTW) and walking/running in slopes (W/R). We further divided the participants in three age groups. 7–9 years (young group) (N = 173), 19–45years (middle group) (N = 134) and 46–80 years (old group) (N = 31). The correlation (Pearson) for the two fine-motor tasks was: young group (7–9), r = 0.179 (p<.05), middle group (19–45), r = .504 (p<.01) and old group (46–80), r = .620 (p<.01) (see fig. 2). The correlation for the two gross-motor tasks were as following in the respective groups; young group (7–9), r = 0.140 (p>.05), middle group (19–45), r = 0.230 (p<.05), old group (46–80), r = 0.845 (p<.01) (see fig. 3). Using Fischer r-to-z transformation showed a significant between group differences in correlation coefficients between young group and old group in both fine motor tasks and gross motor tasks.

## Discussion

This study aimed to explore whether the patterns of lifespan development established by cognitive research are also evident in the motor domain. Another aim was to investigate whether correlations between different motor tasks increases with age.

In the following, each question will be dealt with successively.

### Motor performance across the life-span

Motor performance increased from childhood (7–9) to young adulthood (19–25) and decreased from young adulthood (19–25) to old age (66–80), mirroring the results from cognitive studies [6], [38].

The oldest group (66–80) performed similar to the children (7–9). The gradual decrease of performance, both in the cognitive domain and the motor domain from young adulthood suggest that this could be a general pattern of life-span development [6], [38]. An inverted U shaped curve relationship is indicated between white matter volume and age [14], suggesting that changes in brain structure could be linked to these patterns. Similar shaped curve is found between motor performance and age in this study. The low white matter volume in both children and the oldest adults could cause decreased processing speed [39] and thus be an explanation for the similarity in motor performance.

### Correlation between motor tasks increases with age

Correlations between the two fine motor tasks and the two gross motor tasks were low in the young group (7–9) and were higher for the older age groups (middle group, old group). The finding of increasing correlations between tasks from childhood to early adulthood actually contradicts the age-differentiation hypothesis during early development [40]. This hypothesis, ‘predicts a decrease in the variance accounted for by g (general ability) from childhood to adolescence and the corresponding increase in the number and importance of specific factors’ [41, p. 1525–1532]. The de-differentiation hypothesis claim that the opposite phenomenon is predicted from early maturity to senescence i.e. increase in the importance of general ability and a decrease in the number and importance of the remaining abilities are expected [41]. Our findings are supported by evidence that correlations among different cognitive measures and between cognitive and sensory measures tend to increase with age [35], [36], [42].

It is plausible that this finding occurs as a result of structural changes in the brain. Grey matter has been showed to decline with increasing age [10] and the reduction seems to be nonlinear [14]. Increased correlations between motor tasks with age could also be explained by principles suggested by Edelman [17], [18]. Children have more neurons compared to adults and thus probably also a larger amount of neural groups. At the same time the volume of white matter is low [14], suggesting that at this time of life (childhood), the interconnection between the units of neurons are not strong. Over time, activated neurons form neural groups with other neurons and the connections between them increases. As a result, correlations between performances on similar motor tasks in early development could be expected to be low. This has earlier been indicted in a study on 4-year-old children. Haga et al. [43] found low inter-correlation among eight different motor tasks from the Movement ABC test battery. They argued that it is possible that the process of motor skill learning is specific [44], [45]. This is in line with Sporns and Edelman [46] who argued that training specific tasks will strengthen the neural connections (synapsis) involved in that particular task thus making this behavior more probable to be executed next time.

With increasing age, fewer neural groups are available, leading to activation of the same neural groups although executing different kinds of tasks. This could explain the increasing correlations in older individuals [12]. A decreased number of neural groups would lay a higher strain on the neural groups still operating. To uphold the same analyzing power more neural groups would have to be recruited and thus task-specific activations would be distributed over a larger area. This could explain why ‘high performing’ elderly adults have a larger area of activation in some fMRI studies than younger adults [12], [13], [42].

A possible reason for the reduction in grey matter and white matter in old age is that the brain is an efficient system. According to Neural Darwinism, neurons that are activated survive [17], [18]. In childhood there is an abundance of neurons and possible neural groups which means that there is much room for plasticity, or learning. The child is learning different skills and these skills would be accompanied with structural changes in the brain. With learning and formation of neural groups, less efficient neural groups and neurons die. To increase survival, neural groups would benefit from activation from other neural groups and thus connect with them. The neurons that become less efficient would decay. This could explain why grey matter is reduced, why correlations increase, and why a larger area of the cortex is activated among old adults. A consequence of this line of thinking would be that the principle of ‘use it or lose it’ becomes increasingly important in older adults [47]. It has been shown that individuals that are older have less behavioral plasticity compared to younger individuals [1], additionally, plasticity in the nervous system is reduced [48]. However, although most older adults show some tissue loss over time, there is substantial variability in the magnitude of this change [11]. Results also indicate a trend toward slower rates of brain atrophy in individuals who remain medically and cognitively healthy [11], suggesting that age is not the only defining factor in age related brain changes.

This study had limitations that need to be addressed in future work. A small sample size in the oldest age groups (45–55, 56–65, 66–80), and the use of cross-sectional design vs a longitudinal can be considered as limitations.

### Implications

Our understanding of the principles of life span development can help us to facilitate ability and performance in people that we provide intervention for. If the maintenance and formation of neural groups are experience –dependent, older individuals would benefit from being active. The broadly defined term activity incorporates both physical activity and cognitive activities. As a result of this approach would be that the principle of ‘use it or lose it’ becomes gradually more critical in older adults [42]. Children and adults would almost certainly benefit from task specific training [40]. Task specific training and repetition would probably increase the probability of neural group formation and should increase the strength of the connections between them.

### Key messages

- The gradual decrease of performance, both in the cognitive domain and the motor domain from young adulthood suggest that this could be a general pattern of life-span development
- Understanding of the principles of life span development can help us to facilitate ability and performance in people that we provide intervention for
- The principle of ‘use it or lose it’ becomes gradually more critical in older adults
- Children and adults would almost certainly benefit from task specific training
- The theory of Neural Darwinism can be used as a framework to explain why structural changes occur.
